# Relationship Between Arterial Stiffness Index, Pulse Pressure, and Magnetic Resonance Imaging Markers of White Matter Integrity: A UK Biobank Study

**DOI:** 10.3389/fnagi.2022.856782

**Published:** 2022-06-21

**Authors:** Atef Badji, Julien Cohen-Adad, Hélène Girouard

**Affiliations:** ^1^NeuroPoly Lab, Institute of Biomedical Engineering, Polytechnique Montréal, Montréal, QC, Canada; ^2^Functional Neuroimaging Unit, Centre de Recherche de l’Institut Universitaire de Gériatrie de Montréal, Université de Montréal, Montréal, QC, Canada; ^3^Department of Neurosciences, Faculty of Medicine, Université de Montréal, Montréal, QC, Canada; ^4^Department of Pharmacology and Physiology, Faculty of Medicine, Université de Montréal, Montréal, QC, Canada; ^5^Groupe de Recherche sur le Système Nerveux Central, Montréal, QC, Canada; ^6^Centre Interdisciplinaire de Recherche sur le Cerveau et l’Apprentissage, Montréal, QC, Canada; ^7^Mila - Quebec AI Institute, Montréal, QC, Canada; ^8^Groupe de Recherche Universitaire Sur le Médicament (GRUM), Montréal, QC, Canada

**Keywords:** arterial stiffness index (ASI), pulse pressure (PP), UK Biobank, white matter, MRI

## Abstract

**Background:**

Alzheimer’s disease and dementia in general constitute one of the major public health problems of the 21st century. Research in arterial stiffness and pulse pressure (PP) play an important role in the quest to reduce the risk of developing dementia through controlling modifiable risk factors.

**Objective:**

The aim of the study is to investigate the association between peripheral PP, arterial stiffness index (ASI) and brain integrity, and to discover if ASI is a better predictor of white matter integrity than peripheral PP.

**Materials and Methods:**

17,984 participants 63.09 ± 7.31 from the UK Biobank were used for this study. ASI was estimated using infrared light (photoplethysmography) and peripheral PP was calculated by subtracting the diastolic from the systolic brachial blood pressure value. Measure of fractional anisotropy (FA) was obtained from diffusion imaging to estimate white matter microstructural integrity. White matter hyperintensities were segmented from the combined T1 and T2-weighted FLAIR images as a measure of irreversible white matter damage.

**Results:**

An important finding is that peripheral PP better predicts white matter integrity when compared to ASI. This finding is consistent until 75 years old. Interestingly, no significant relationship is found between either peripheral PP or ASI and white matter integrity after 75 years old.

**Conclusion:**

These results suggest that ASI from plethysmography should not be used to estimate cerebrovascular integrity in older adults and further question the relationship between arterial stiffness, blood pressure, and white matter damage after the age of 75 years old.

## Introduction

Alzheimer’s disease and dementia in general is one of the major public health problems of the 21st century (Wiesmann et al., 2013; World Health Organization, 2015), yet there is currently no cure nor disease-modifying treatment for it. This stresses the need to find ways to reduce the risk of developing dementia through controlling modifiable factors (Baumgart et al., 2015). Vascular risk factors, such as hypertension and arterial stiffness have been associated with the pathogenesis of dementia, in particular Alzheimer’s disease and vascular dementia through their impact on the white matter (Singer et al., 2014; Badji et al., 2019, 2020).

White matter lesions described as white matter hyperintensities by T2-weighted or fluid-attenuated inversion recovery (FLAIR), have consistently been shown to be associated with arterial stiffness of large arteries (Longstreth et al., 1996; Tsao et al., 2013; Maillard et al., 2016). Studies using diffusion imaging highlight that arterial stiffness alters the microstructural integrity of three vulnerable white matter tracts (corpus callosum, internal capsule, and corona radiata) prior to irreversible white matter lesions (Badji et al., 2019).

Arterial stiffness increases with age. Indeed, as we age, our large elastic vessels undergo progressive luminal dilatation, thickening of the arterial wall, increased deposition of collagen, and combined fragmentation and degeneration of elastin which reduce their capacity for dampening blood pulsatility arising from the heart during each contraction (Wiesmann et al., 2013). As a result, arterial stiffness leads to an increase of the pulse wave propagation which escalates systolic blood pressure (SBP) (Laurent et al., 2005; Pase, 2012). During aging, diastolic blood pressure (DBP) is also known to decrease. Arterial stiffness contributes therefore to the increase of pulse pressure (PP) as it reflects the difference between SBP and DBP (Kaess et al., 2012).

Increased PP and arterial stiffness are strongly correlated. However, they are also considered independent measures of vascular aging (Franklin et al., 1999; Mitchell et al., 2010). Indeed, on the one’s hand, PP has been shown to be strongly associated with outcomes such as coronary heart disease (CHD), and cardiovascular events in hypertensive patients (Madhavan et al., 1994), elderly (Vaccarino et al., 2000), and the general population (Glasser et al., 2014). On the other hand, arterial stiffness as measured by carotid-femoral pulse wave velocity (cfPWV), which is considered the gold standard measure of arterial stiffness has been shown not only to be related to CHD (Boutouyrie et al., 2002), stroke (Vlachopoulos et al., 2010) but also to risk factors for dementia (Iulita et al., 2018) such as atherosclerosis (Fernandes et al., 2008), hypertension (Laurent et al., 2006), metabolic syndrome (Laurent et al., 2006) and diabetes mellitus (Laurent et al., 2006).

While cfPWV provides excellent prognostic value in adults, it is cumbersome due to the need for specialized equipment and remains mostly utilized in research nowadays (Matsui et al., 2004; Nichols, 2005). Conversely, the arterial stiffness index (ASI) estimates arterial stiffness using infrared light (photoplethysmography) to record the volume waveform of the blood in the finger in 10–15 s (Chowienczyk et al., 1999; Millasseau et al., 2000, 2002). It is therefore a fast, inexpensive, simple-to-use and therefore convenient measure of arterial stiffness that does not require specific expertise (Elgendi, 2012).

Recently, Said et al. (2018) investigated the association between vascular aging as indicated by ASI and peripheral PP with cardiovascular disease (CVD) risk factors, CVD events and mortality in 169,613 participants from the UK Biobank. Said et al. (2018) highlighted in their study that peripheral PP has more added value than ASI to improve the risk classification of incident cardiovascular disease. However, this study did not investigate the association between peripheral PP, ASI and brain integrity. Therefore, several questions remain: Is ASI a predictor of white matter integrity? If so, is it a better predictor of white matter integrity than peripheral PP? In this study we investigated the association of vascular aging as indicated by ASI and peripheral PP with magnetic resonance imaging (MRI) markers of white matter integrity.

## Materials and Methods

### Study Participants

The data from the UK Biobank resource were used for the purpose of this study. The UK Biobank study design and population had previously been detailed (Sudlow et al., 2015). In brief, UK Biobank is a large community-based prospective study in the United Kingdom that recruited 502,299 participants aged 40-85 years old with the aim of improving prevention, diagnosis, and treatment of a plethora of illnesses. The study collected detailed phenotype and genotype data, including sociodemographic, lifestyle, clinical diagnosis, treatment genetic, imaging and physiological parameters. UK Biobank has approval from the institutional review boards, namely, the North West Multi-centre Research Ethics Committee for the United Kingdom, from the National Information Governance Board for Health and Social Care for England and Wales, and from the Community Health Index Advisory Group for Scotland^1^. All participants gave informed consent for the study via a touch-screen interface that required agreement for all individual statements on the consent form as well as the participant’s signature on an electronic pad^2^. The present study is restricted to a subsample of United Kingdom participants with information of ASI, blood pressure, diffusion tensor imaging (DTI) and white matter hyperintensities volumes (WMHV). Those with self-reported conditions listed in SM1 were excluded which led to a total of 20,742 participants included in the study. The research reported here was conducted using the UK Biobank Resource under Application Number 54531.

### Cardiovascular Measurements

#### Arterials Stiffness Index

Pulse wave velocity was measured using the PulseTrace PCA2 (CareFusion, San Diego, CA, United States) which uses finger photoplethysmography to record a 10–15 s pulse waveform on the index finger of the participant’s dominant hand (Collins and UK Biobank Steering Committee, 2007). The pulse waveform comprises a systolic peak and second diastolic peak, and the transit time [peak-to-peak time (PPT)] between the 2 peaks is related to the time it takes for the pulse wave to travel through the peripheral arterial tree (Millasseau et al., 2002; Gunarathne et al., 2008). This path length is proportional to a person’s height (h), enabling the calculation of an index of large artery stiffness using the formula ASI = h/PPT, expressed in m/s (Gunarathne et al., 2008).

#### Blood Pressure Measurements and Pulse Pressure

SBP and DBP were measured twice by trained nurses after the participant had been at rest for at least 5 min in the seated position using an automated blood pressure device [Omron 705 IT electronic blood pressure (BP) monitor; OMRON Healthcare Europe B.V. Kruisweg 577 2132 NA, Hoofddorp, Netherlands], or manually using a sphygmomanometer with an inflatable cuff in combination with a stethoscope. For the purpose of the study, we first calculated the mean SBP and DBP from the two available automated BP measures for each participant. Then, since automated devices tend to measure higher SBPs than manual sphygmomanometers, we adjusted both SBP and DBP that were measured using the automated device using algorithms by Stang et al. (2006) as done recently by Said et al. (2018) for the UK Biobank data. The peripheral PP was calculated by subtracting the DBP from the SBP brachial values.

### Brain Magnetic Resonance Imaging Analysis

#### Data Acquisition

All brain MRI data were acquired on a Siemens Skyra 3T scanner using a standard Siemens 32-channel head coil according to a freely available protocol^3^.

Diffusion-weighted images were acquired using a spin-echo echo-planar sequence encoded with 5 *b* = 0 s/mm^2^ (plus an additional 3 blip-reversed *b* = 0 s/mm^2^), 50 *b* = 1,000 s/mm^2^ and 50 *b* = 2,000 s/mm^2^ diffusion-weighted volumes, for a total of 100 distinct diffusion-encoding directions. A multiband acceleration factor of 3 was used for acquisitions, spatial resolution = 2.0 mm isotropic, TR = 3,600 ms, TE = 92.00 ms. T2-weighted FLAIR volumes were acquired in sagittal orientation at 1.05 × 1 × 1 mm resolution with the 3D space optimized readout (Mugler, 2014) with the following parameters: TR = 5,000 ms, TE = 395 ms.

#### Diffusion Weighted imaging Processing

Imaging-derived data generated by an image-processing pipeline developed and run by the UK Biobank team were used in this study (Alfaro-Almagro et al., 2018). Briefly, preprocessing of diffusion data included Eddy current distortion correction, head motion correction, and outlier slices correction using FSL’s Eddy tool (Andersson and Sotiropoulos, 2015, 2016), prior to gradient distortion correction. Next, fractional anisotropy (FA) was calculated by fitting a diffusion tensor model to the pre-processed single-shell Diffusion weighted imaging (DWI) (*b* = 1,000 s/mm^2^) data using FSL’s DTIFIT (Nir et al., 2017).

The FA maps were used in FSL’s Tract-Based Spatial statistics (TBSS) (Smith et al., 2006) and TBSS-derived measures were computed by averaging the skeletonized image of each FA map within a set of 48 standard-space tract masks defined by the JHU white matter atlas (ICBM-DTI-81) (Mori et al., 2008). For the purpose of the study the mean FA in the corpus callosum (CC), the internal capsule (IC) and the corona radiata (CR) were used.

White matter hyperintensities (WMH) were automatically segmented from the combined T1 and T2-weighted FLAIR images using the Brain Intensity Abnormality Classification Algorithm tool (Griffanti et al., 2016). An automated pipeline was used to delineate white matter hyperintensities from the FLAIR images. The full details of the image processing and QC pipeline are available in an open-access article (Alfaro-Almagro et al., 2018). The WMHs volumes were expressed as the percentage of intracranial volume and then natural log-transformed.

### Statistical Analysis

Prior to analysis, we tested the presence of outliers in each variable of interest and removed 2,758 participants in total ASI, WMHV, FA in CC, IC and CR data using the 1.5 interquartile rule. Therefore in this study, we only considered the remaining 17984 participants. The association between vascular risk factors (e.g., increased in peripheral PP or in ASI) and changes in the white matter and cognitive decline is complex, and largely mediated by mixed cerebrovascular and neurodegenerative lesions (Qiu and Fratiglioni, 2015; Wang et al., 2017). These associations have been found to be stronger when multiple vascular risk factors are present in mid-life (40–59 years of age), especially if left untreated (Launer et al., 1995; Kivipelto et al., 2001; Yaffe et al., 2014; Qiu and Fratiglioni, 2015). These associations are in contrast less certain when these vascular risk factors occur in later life (>75 years of age) (Qiu et al., 2005; Novak and Hajjar, 2010; Anstey et al., 2011; Tolppanen et al., 2012). For that reason, we divided our participants into 4 groups based on their age G1 (45–55 years), G2 (55–65 years), G3 (65–75 years), and G4 (≥75 years).

To test the hypothesis that ASI is a better predictor of white matter integrity than peripheral PP, a stepwise linear model was performed. For this analysis, each MRI metric was treated as a dependent variable and all the following variables were considered as independent variables: age, sex, peripheral PP, and ASI (Table 2). To better understand the relationship between vascular aging and white matter damage across the age span, we then looked at the relationship between either peripheral PP or ASI with our MRI markers of white matter integrity across different age brackets. Scatter plots between age and peripheral PP, ASI, FA in the CC, and WMHV, respectively, further help to interpret the results (SM2).

All statistical tests and figures except the stepwise linear regression were done with R version 3.5.2. The stepwise linear regression was done with SPSS (IBM SPSS 25, Statistics, Chicago, IL, United States).

To promote reproducibility, the data and code to perform all statistical analyses and figures are publicly available at https://github.com/atefbadji/ASI_PP_MRI_UKBiobank.

## Results

This study included 17,984 participants, mean age 63.09 ± 7.31 years, of which 9,311 are female. Table 1 presents the descriptive statistics for the sample as well as for subsample based on age bracket. Differences between groups in demographic and clinical variables were analyzed using an analysis of variance (ANOVA) or Chi-squared (χ^2^) tests for continuous or categorical variables, respectively. One can note that all comparisons were significant among groups (*p* < 0.001). We even performed additional groupwise analysis and found almost all pairwise comparisons to be significant for all variables. The only groupwise comparison that were not significant were the following: G1 vs. G2 (*p* = 0.82) and G3 vs. G4 (*p* = 0.07) for BMI, G1 vs. G2 (*p* = 0.09) for DBP, G2 vs. G4 (*p* = 0.32) and G3 vs. G4 (*p* = 0.34) for ASI and finally G3 vs. G4 (*p* = 0.36) for FA IC. However, due to the very large sample size, one can wonder about the statistical relevance from a clinical point of view. These results are in line with the literature but outside the scope of the present study. They are therefore not discussed in the present study.

**TABLE 1:** Characteristics of all participants.

	All participants	Between 45 and 55 years	Between 55 and 65 years	Between 65 and 75 years	Over 75 years	*p*-value
Women/men (N)	9,311/8,673 (17,984)	1,652/1,363 (3,015)	3,986/3,320 (7,306)	3,365/3,510 (6,875)	308/480 (788)	**<2.2e-16**
Age (years)	63.09 ± 7.31	52.14 ± 1.94	60.25 ± 2.88	69.34 ± 2.67	76.86 ± 1.47	**<2.0e-16**
BMI (kg/m^2^)	26.17 ± 4.12	26.34 ± 4.40	26.30 ± 4.26	26.00 ± 3.91	25.65 ± 3.44	**7.42e-8**
**Cardiovascular measures**						
SBP (mmHg)	131.53 ± 16.61	124.80 ± 15.36	129.78 ± 16.23	135.54 ± 16.28	138.45 ± 15.89	**<2.0e-16**
DBP (mmHg)	78.93 ± 8.24	79.15 ± 8.42	79.35 ± 8.23	78.54 ± 8.12	77.45 ± 8.26	**<1.92e-16**
PP (mmHg)	52.60 ± 12.36	45.65 ± 9.93	50.43 ± 11.30	55.99 ± 12.20	60.99 ± 12.58	**<2.0e-16**
ASI (m/s)	9.37 ± 2.67	8.96 ± 2.46	9.35 ± 2.62	9.55 ± 2.76	9.45 ± 2.76	**<2.0e-16**
**MRI measure**						
FA in CC	0.745 ± 0.021	0.751 ± 0.019	0.747 ± 0.020	0.741 ± 0.021	0.734 ± 0.022	**<2.0e-16**
FA in IC	0.630 ± 0.018	0.633 ± 0.017	0.630 ± 0.017	0.628 ± 0.018	0.627 ± 0.018	**<2.0e-16**
FA in CR	0.479 ± 0.019	0.486 ± 0.018	0.482 ± 0.019	0.475 ± 0.019	0.469 ± 0.019	**<2.0e-16**
WMHV	3095.41 ± 2422.66	1693.63 ± 1466.11	2593.85 ± 2043.15	3992.16 ± 2579.82	5285.21 ± 2704.12	**<2.0e-16**

*Values are means ± standard deviation. SBP, systolic blood pressure; DBP, diastolic blood pressure; PP, pulse pressure; FA in CC and FA in CR are fractional anisotropy of the corpus callosum, internal capsule and corona radiata, respectively; WMHV, white matter hyperintensities volume. Bold values are significant values after correction for multiple comparison.*

**TABLE 2:** Result of stepwise entry linear model based on a priory hypothesis on the influence of age, sex, and vascular risk factors on white matter integrity.

Dependent variable = FA CC				
Name	Independent variables	*R* ^2^	*p*	*F* change *p*
1	Age + Sex	0.050	<0.001	N/A
2	Age + Sex + PP	0.051	<0.001	<0.001
**Dependent variable = FA IC**				
Name	Independent variables	*R* ^2^	*P*	*F* change *p*
1	Age + Sex	0.031	<0.001	N/A
2	Age + Sex + PP	0.032	<0.001	<0.001
**Dependent variable = FA CR**				
Name	Independent variables	*R* ^2^	*p*	*F* change *p*
1	Age + Sex	0.068	<0.001	N/A
2	Age + Sex + PP	0.069	<0.001	<0.001
**Dependent variable = WMHV**				
Name	Independent variables	*R* ^2^	*p*	*F* change *p*
1	Age + Sex	0.184	<0.001	N/A
2	Age + Sex + PP	0.189	<0.001	<0.001
3	Age + Sex + ASI	0.190	<0.001	<0.001

*Two cardiovascular risk factors were fed to each stepwise linear model: PP and ASI. These models aimed to find which cardiovascular risk factors among PP and ASI best predict the fractional anisotropy (FA) of the corpus callosum (CC), internal capsule (CI), corona radiata (CR) and the white matter hyperintensity volume (WMHV). Fixed covariates were age and sex. R^2^ of the significant models are reported as well as the overall p-value of the model and the significant F changes when comparing with previous model.*

To answer our hypothesis that ASI is not only a predictor of white matter integrity but also a better one than peripheral PP, we performed a stepwise linear regression (Table 2). In Table 2, model FA CC_1 revealed that age and sex alone predict FA in the CC (*p* < 0.001). This analysis indicated an *R*^2^ of 0.05, which means that age and sex accounted for about 5% of the total variance of FA CC. Interestingly, model FA CC_2 highlights that peripheral PP is found to be the best predictor of FA CC in comparison with ASI. Indeed, the contribution of peripheral PP on top of age and sex was found to account for significantly more variance than the previous model (*F* change at *p* < 0.001). The overall model of FA CC_2 (age, sex, and peripheral PP) was also found to be a significant predictor of FA CC_2 (*p* < 0.001). This analysis indicated an *R*^2^ of 0.051 which means that age, sex, and peripheral PP accounted for 5.1% of the total variance of FA CC. Similar results have been found for all DTI metrics of interest (FA IC and FA CR, Table 2). In particular, age, sex, and peripheral PP were found to account for 6.9% of the total variance of FA CR. Table 2 also highlights that age and sex predict WMHV independently (*p* < 0.001). This analysis indicated and *R*^2^ of 0.184 which means that age and sex accounted for about 18.4% of the total variance of WMHV.

Interestingly, model WMHV_2 highlights that peripheral PP is found to be the best predictor of WMHV in comparison to ASI. Indeed, the contribution of peripheral PP on top of age and sex was found to account for significantly more variance than the previous model (*F* change at *p* < 0.001). The overall model of WMHV (age, sex, and peripheral PP) was also found to be a significant predictor of WMHV (*p* < 0.001). This analysis indicated an *R*^2^ of 0.189 which means that age, sex, and peripheral PP accounted for 18.9% of the total variance of WMHV. Moreover, the contribution of ASI on top of age, sex, and peripheral PP was found to account for significantly more variance than the previous model (*F* change at *p* < 0.001). The overall model of WMHV (age, sex, peripheral PP, and ASI) was found to be a significant predictor of WMHV (*p* < 0.001). This analysis indicated an *R*^2^ of 0.190 which means that age, sex, peripheral PP, and ASI accounted for 19% of the total variance of WMHV.

**FIGURE 1:**
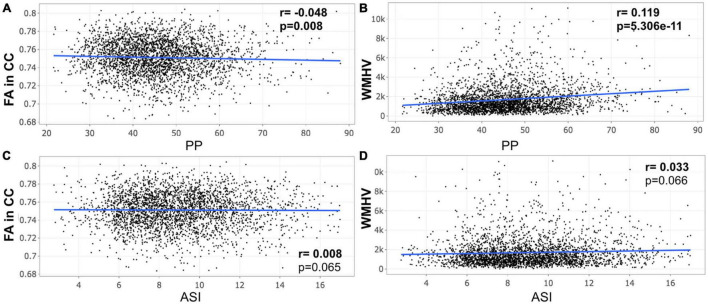
Scatter plot for partial correlation analysis of either pulse pressure (PP) or arterial stiffness index (ASI) with the fractional anisotropy of the corpus callosum (FA in CC) or the white matter hyperintensity volume (WMHV) in 3,015 participants between 45 and 55 years old. Significant *p*-values are in bold (adjusted threshold with FDR was 0.025). Covariates included is sex. An additional analysis was performed with both age and sex as covariates showing similar significant results: **(A)** *p* = 0.012; **(B)** *p* = 2.65e-09.

**FIGURE 2:**
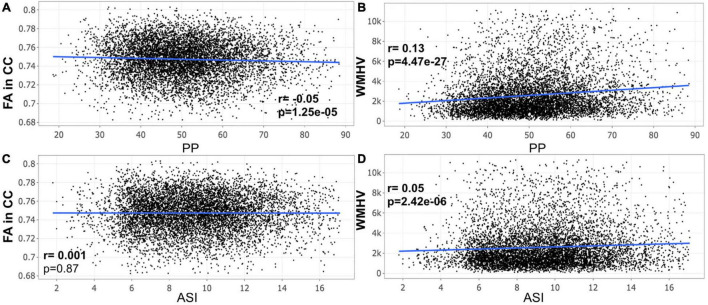
Scatter plot for partial correlation analysis of either pulse pressure (PP) or stiffness Index (ASI) with the fractional anisotropy of the corpus callosum (FA in CC) or the white matter hyperintensity volume (WMHV) in 7,306 participants between 55 and 65 years old. Significant *p*-values are in bold (adjusted threshold with FDR was 0.0375). Covariates included sex. An additional analysis was performed with both age and sex as covariates showing similar significant results: **(A)** *p* = 5.2e-04; **(B)** *p* = 7.38e-17; **(D)**, *p* = 2.75e-05.

**FIGURE 3:**
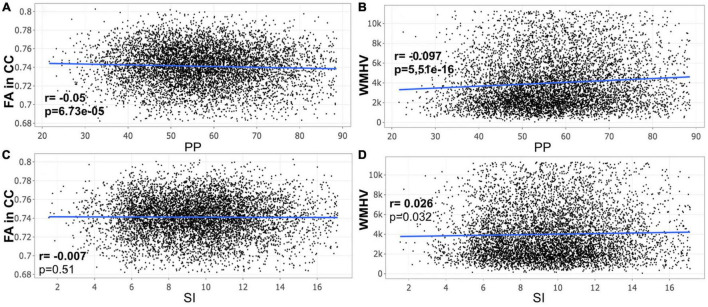
Scatter plot for partial correlation analysis of either pulse pressure (PP) or stiffness Index (ASI) with the fractional anisotropy of the corpus callosum (FA in CC) or the white matter hyperintensity volume (WMHV) in 6,875 participants between 65 and 75 years old. Significant *p*-values are in bold (adjusted threshold with FDR was 0.0375). Covariates included sex. An additional analysis was performed with both age and sex as covariates showing similar significant results: **(A)** *p* = 2.03e-05; **(B)** *p* = 6.73e-33; **(D)** *p* = 1.54e-06.

**FIGURE 4:**
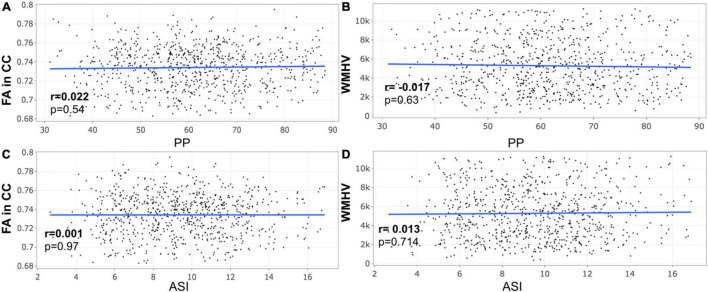
Scatter plot for partial correlation analysis of either pulse pressure (PP) or stiffness Index (ASI) with the fractional anisotropy of the corpus callosum (FA in CC) or the white matter hyperintensity volume (WMHV) in 788 participants over 75 years old. Significant *p*-values are in bold (adjusted threshold with FDR was 0.0125). Covariates included is sex. An additional analysis was performed with both age and sex as covariates showing similar results.

A closer look at the association between peripheral PP and ASI with our MRI metrics of interest revealed that peripheral PP was negatively associated with FA in CC (*r* = −0.048, *p* = 0.008) and positively WMHV (*r* = 0.119, *p* = 5.306e-11) in participants aged 45–55 years old (Figure 1). The same relationship was found in participants aged between 55 and 65 (Figure 2) and 65 and 75 years old (Figure 3). However, ASI was not found to be associated with neither FA in CC nor WMHV in any group of participants (Figures 1–4) with the exception of a positive association found between ASI and WMHV in participants aged 55–65 years old (*r* = 0.05, *p* = 2.42e-06). Interestingly, in participants over 75 years old, we did not find a significant relationship between either peripheral PP or ASI with MRI metrics (Figure 4).

## Discussion

In this study, we investigated the relationship between ASI and peripheral PP with white matter integrity as assessed by FA metric and WMHV volumes in participants of the UK Biobank. An important finding is that peripheral PP better predicts white matter integrity when compared to ASI measured with plethysmography. We found that an increase in peripheral PP was associated with a lower FA and increased WMHV. This finding was consistent until 75 years old. Interestingly, no significant relationship was found between neither peripheral PP nor ASI and white matter integrity after 75 years old, which may question the nature of the relationship between markers of vascular aging and white matter damage in individuals over 75 years old.

### Pulse Pressure Is a Better Predictor of White Matter Integrity Than Arterial Stiffness Index in Participants Aged 45–75 Years Old

Several studies have previously compared different markers of arterial stiffness and vascular aging on the incidence of cardiovascular events, in particular cfPWV and PP. cfPWV has been established as the gold standard measure of arterial stiffness and a stronger predictor of cardiovascular events than peripheral PP, not only in older adults but also in individuals with different conditions such as renal failure, atrial fibrillation, and heart failure (Laurent et al., 2001; Rahman, 2017; Niiranen et al., 2019). Peripheral PP has also been associated with cardiovascular outcomes (Madhavan et al., 1994; Benetos et al., 1997; Mitchell et al., 1997), however, such observation has been attributed to the interplay between peripheral PP and arterial stiffness.

Beyond its effects on cardiovascular events, cfPWV has been also shown to better predict cognitive decline than measures of BP (Marfella and Paolisso, 2016). The emerging MRI literature has often highlighted a relationship between arterial stiffness, WMHs and white matter microstructure (Shrestha et al., 2009; Rosano et al., 2013; Singer et al., 2014; Badji et al., 2019). In particular, DTI studies looking at the effect of cfPWV on the white matter microstructure showed consistently that several white matter tracts are particularly vulnerable to increased arterial stiffness, among which is the CC, a key white matter tract altered in AD (Badji et al., 2019). Interestingly, cfPWV was recently found to better predict the microstructural integrity of the CC when compared with measures of 24 h SBP, 24 h PP and AIx in participants between 65 and 75 years old (Badji et al., 2020). However, to the best of our knowledge, no other study has yet compared different markers of arterial stiffness on brain integrity, nor has included measures of ASI before the present study.

In the emerging literature, the correlation between ASI and cfPWV has been found convincing (*r* = 0.65, *p* < 0.001) (Millasseau et al., 2002). The relationship between ASI, age and BP was even found similar to that between cfPWV, age and BP which further supported the concept that ASI and cfPWV are influenced by similar factors (Millasseau et al., 2002). Studies looking at the effect of ASI on brain integrity would therefore add value to the already published literature in terms of similitude and differences of such markers. This will in turn hopefully help clinicians in their choice of markers of vascular aging with respect to the outcome of interest.

Our study presents the first evidence that peripheral PP better predicts white matter integrity compared to ASI in participants of the UK Biobank younger than 75 years old. This finding implies that ASI from plethysmography may not be a reliable measure of vascular aging in older adults, and may therefore not be used to estimate cerebrovascular integrity in older adults.

A number of reasons can explain this result. The contour of the pulse waveform is essentially determined by characteristics of the heart such as ventricular ejection and those of large arteries which are altered with increasing age (Avolio et al., 1983; Millasseau et al., 2002; Alty et al., 2007; Russo et al., 2011). In older individuals, the systolic and diastolic peaks in the contour of the arterial waveform become difficult to identify (McVeigh et al., 1999; Alty et al., 2007). This was already demonstrated by McVeigh et al. (1999) who measured the arterial waveform shapes in a group of people of different ages. Increased arterial stiffness in older adults elevates arterial pressure wave propagation, causing the reflected wave to arrive back at the aorta during the systolic rather than the diastolic phase of the cardiac cycle, thereby escalating the SBP and contributing to a widening of PP (Laurent et al., 2005; Vlachopoulos et al., 2011; Pase et al., 2012). The time between the peaks of forward and reflected waves assessed using plethysmography appeared thereby reduced in older subjects (Millasseau et al., 2002). In addition, studies looking at age-related changes in shape characteristics in individual fingers and toes using frequency spectrum analysis (Oliva et al., 1976; Sherebrin and Sherebrin, 1990) showed that there is a general reduction in the harmonic components of the pulse in older subjects (Shirouzu et al., 1998) which makes it further difficult to estimate ASI using plethysmography technique in older adults.

### Arterial Stiffness Index, Pulse Pressure, and Brain Integrity After 75 Years Old

As mentioned in the introduction, peripheral PP is determined by brachial SBP and DBP, and the relative contribution of these is a function of age. In young adults, both DBP and SBP increase, whereas in the elderly SBP increases and DBP reduces with age, which escalates PP (Wills et al., 2011). The association among higher SBP, mean BP, DBP, peripheral PP, and WMH is consistent in many previous studies during adulthood (Singer et al., 2014; Badji et al., 2019). Likewise, the association between high BP in middle age (age 40–64 years) and increased risk for vascular dementia is well established (Kennelly et al., 2009; Launer et al., 2010; Ninomiya et al., 2011). However, studies looking at these associations at an older age are sparse and there is no consensus on the association between BP and dementia in individuals aged 75 and above (Peng et al., 2017). In addition, information about the impact of BP and arterial stiffness measures on white matter integrity in people over 75 years old is still lacking.

Nevertheless, a recent study investigated the association between BP measures longitudinally, the internal carotid arteries (ICA) blood flow velocity parameters, and age-related WMH in a well-characterized 694 community-dwelling cohort of older adults over 70 years old (Aribisala et al., 2014). Results from this study showed that no association was found between peripheral PP and WMH measures (both WMH volume or Fazekas), agreeing well with the results of the present study. Looking at the results in detail, Aribisala et al. (2014) also showed that the size of WMH at the age of 73 years was weakly associated with mean BP and DBP at the age of 70 years old. Similar but even weaker associations were seen between WMH and BP at the age of 73 years (Aribisala et al., 2014). In addition, Peng et al. (2017) reported no association between BP levels nor peripheral PP at the age of 70–75 years and the incidence of vascular dementia.

Altogether, these results could be explained by different reasons. Indeed, both SBP and DBP can reduce at an older age. This phenomenon has a multifactorial etiology such as frailty, polymedication, improper/non-adjusted medication, heart failure, kidney failure, and so on and so forth. The integrity of the brain has a critical dependence on a nearly impeccable vascular supply, it is therefore not excluded that a decline in BP happens as a result of brain damage in the pathogenesis of dementia, just as much as the multifactorial-related decline in BP can further damage cerebrovascular integrity leading to dementia. Such a decline in BP could weaken the association between increased BP levels, white matter changes, and the incidence of vascular dementia later in life (Skoog et al., 1996). Although individuals with any dementia diagnosis or neurological condition were excluded from the present study, some of our participants over 75 years old may be in the early stage of dementia, before clinical symptoms appear. This could explain the significant association found between decreased peripheral PP and increased WMHs in individuals between 65 and 75 years, and the non-significant association between peripheral PP and either FA or WMHs in participants over 75 years old.

Another issue with BP measures in general in late life is the lack of information regarding the dose of exposure (and untreated exposure) in most studies, including this one. Those with a new-onset PP increase may not have an immediate risk of brain injury but those with prolonged PP elevation that continues into late life are likely at very high risk. Effects of treatment only compound these dose-dependent associations that are typically unmeasured.

### Strengths and Limitations

The strength of this study lies in the use of a large community-dwelling cohort from the United Kingdom and well-validated imaging-derived data used across all researchers accessing the UK Biobank data. To the best of our knowledge this is the first study comparing different markers of arterial stiffness on brain integrity including ASI from plethysmography. Including comparisons of drug-naive participants and those on antihypertensive treatments would have been interesting. Unfortunately, none of the women included in the present study presented information about antihypertensive medication history, therefore we could not have included it. Likewise, excluding participants with heart conditions such as valve diseases, cardiac arrhythmias or heart failure and participants with renal or hepatic failure was not possible due to the lack of information in the UK Biobank database. Finally, although we did not find any relationship between either peripheral PP or ASI and white matter integrity after 75 years, we must acknowledge that we had less participants (*n* = 788) compared to other groups (*n* = 3,015–17,984). Fewer subjects may imply less power to detect such association. In this study, we looked for the first time at the relationship between peripheral PP, ASI and white matter microstructure after the age of 75 years. Although our finding may question the nature of the relationship between markers of vascular aging and white matter damage in individuals over 75 years old, it certainly needs to be replicated in a larger study before being able to draw an accurate conclusion.

## Conclusion

This study is the first to investigate the relationship between ASI and peripheral PP with the white matter integrity as assessed by FA metric and WMHV volumes in participants of the UK Biobank. Our results show that peripheral PP predicts white matter integrity better than ASI in participants younger than 75 years old. These results suggest that ASI from plethysmography should not be used to estimate cerebrovascular integrity in older adults. In addition, no significant relationship was found between either peripheral PP or ASI and white matter integrity after 75 years old. A decline of BP in the pathogenesis of dementia could weaken the association between increased BP levels, white matter alteration, and the incidence of vascular dementia at a late stage in life (Skoog et al., 1996).

## Data Availability Statement

The data analyzed in this study is subject to the following licenses/restrictions: UK Biobank is a large-scale biomedical database and research resource, containing in-depth genetic and health information from half a million United Kingdom participants. The database is regularly augmented with additional data and is globally accessible to approved researchers undertaking vital research into the most common and life-threatening diseases. Requests to access these datasets should be directed to https://www.ukbiobank.ac.uk/.

## Ethics Statement

The studies involving human participants were reviewed and approved by the UK Biobank has approval from the Institutional Review Boards, namely, the North West Multi-centre Research Ethics Committee for the United Kingdom, from the National Information Governance Board for Health and Social Care for England and Wales, and from the Community Health Index Advisory Group for Scotland (https://www.ukbiobank.ac.uk/wp-content/uploads/2011/05/EGF20082.pdf). All participants gave informed consent for the study via a touch-screen interface that required agreement for all individual statements on the consent form as well as the participant’s signature on an electronic pad (http://www.ukbiobank.ac.uk/wp-content/uploads/2011/06/Consent_form.pdf). Written informed consent for participation was not required for this study in accordance with the National Legislation and the Institutional Requirements.

## Author Contributions

AB: design of the study, analysis, statistics, writing, submission of the article, and revision of the manuscript. JC-A and HG: design of the study and writing and revision of the manuscript. All authors contributed to the article and approved the submitted version.

## Conflict of Interest

The authors declare that the research was conducted in the absence of any commercial or financial relationships that could be construed as a potential conflict of interest.

## Publisher’s Note

All claims expressed in this article are solely those of the authors and do not necessarily represent those of their affiliated organizations, or those of the publisher, the editors and the reviewers. Any product that may be evaluated in this article, or claim that may be made by its manufacturer, is not guaranteed or endorsed by the publisher.

## Footnotes

1
https://www.ukbiobank.ac.uk/learn-more-about-uk-biobank/about-us/ethics

2
https://www.ukbiobank.ac.uk/media/05ldg1ez/consent-form-uk-biobank.pdf

3
https://www.fmrib.ox.ac.uk/ukbiobank/protocol/V4_23092014.pdf

## Supplementary Material

Supplementary Figure 1 Scatter plots between age and peripheral PP, ASI, FA in the CC and WMHV respectively.
