# Supplementary material for: Relationship Between Arterial Stiffness Index, Pulse Pressure, and Magnetic Resonance Imaging Markers of White Matter Integrity: A UK Biobank Study
Source: Front Aging Neurosci. 2022 Jun 21;14:856782. doi: 10.3389/fnagi.2022.856782 (PMC9252854; doi:10.3389/fnagi.2022.856782)
Supplement: Supplementary file 1 [file Table_1.DOCX]

**Supplemental 1**: **List of clinical diagnosis excluded from the present study**

Current smoking, diabetes, asthma, emphysema, chronic bronchitis, chronic obstructive pulmonary disease, cystic fibrosis, alpha-1 antitrypsin deficiency, sarcoidosis, bronchiectasis, idiopathic pulmonary fibrosis, fibrosing alveolitis/unspecified alveolitis, tuberculosis, silicosis, asbestosis, lung cancer inclusive mesothelioma of the lung, stroke including ischemia, iodine deficiency related thyroid disorder, subclinical iodine deficiency hypothyroidism, other non-toxic goiter, thyrotoxicosis, thyroiditis, other disorders of the thyroid, hypoparathyroidism, hyperparathyroidism and other disorders of the parathyroid gland, Huntington's disease, hereditary ataxia, systemic atrophies primarily affecting central nervous system in diseases classified elsewhere, post-polio syndrome, Parkinson’s disease, secondary parkinsonism, parkinsonism in diseases classified elsewhere, other degenerative diseases of basal ganglia, dystonia, other extrapyramidal and movement disorders, Alzheimer’s disease, other degenerative diseases of nervous system, multiple sclerosis, other acute disseminated demyelination, other demyelinating diseases of central nervous system, epilepsy, status epilepticus, transient cerebral ischaemic attacks and related syndromes, vascular syndromes of brain in cerebrovascular diseases, toxic encephalopathy, rheumatic chorea, rheumatic mitral valves diseases, rheumatic aortic valve diseases, multiple valve diseases, other rheumatic heart diseases, paroxysmal tachycardia, atrial fibrillation and flutter, other cardiac arrhythmias, heart failure, hepatic failure, acute renal failure, chronic renal failure and unspecified renal failure.
