# Supplementary figures and images for: Relationship Between Arterial Stiffness Index, Pulse Pressure, and Magnetic Resonance Imaging Markers of White Matter Integrity: A UK Biobank Study
Source: Front Aging Neurosci. 2022 Jun 21;14:856782. doi: 10.3389/fnagi.2022.856782 (PMC9252854; doi:10.3389/fnagi.2022.856782)

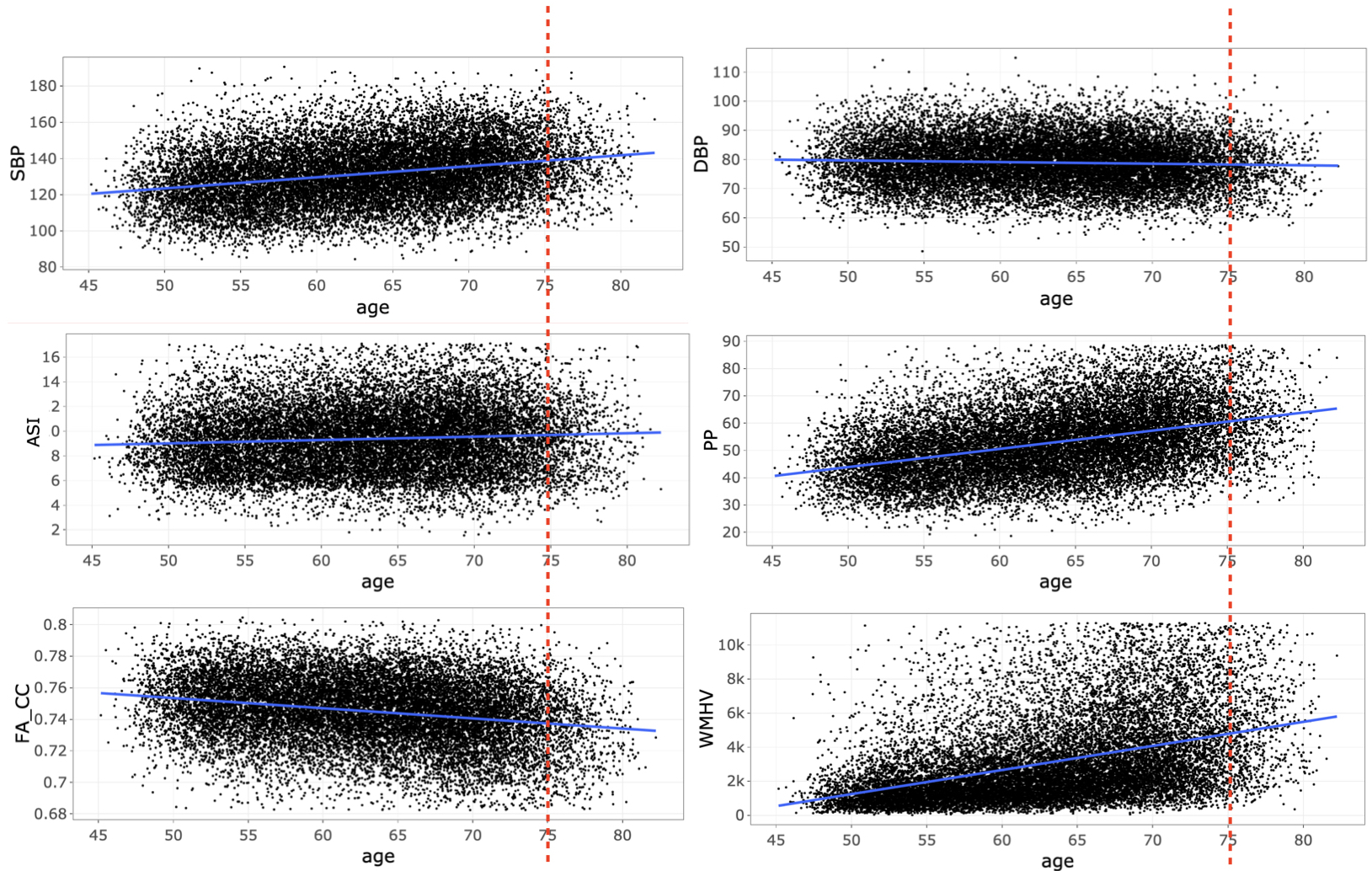

Supplement: Supplementary Figure 1 — Scatter plots between age and peripheral PP, ASI, FA in the CC and WMHV respectively. [file Image_1.JPEG]
